# Beyond the liver: primary appendiceal hydatid disease with disseminated peritoneal, ovarian, and uterine involvement mimicking an appendiceal neoplasm

**DOI:** 10.1093/jscr/rjag1011

**Published:** 2026-11-10

**Authors:** Tilak Dangi, Ranjish Parshaila, Nikhil Agarwal, Dipendra Thakur, Rohit P Yadav

**Affiliations:** Department of General Surgery, Birat Medical College Teaching Hospital, Tankisinuwari, Morang, Biratnagar 56613, Province 1, Nepal; Department of Gastrointestinal Surgery, Birat Medical College Teaching Hospital, Tankisinuwari, Morang, Biratnagar 56613, Province 1, Nepal; Department of Gastrointestinal Surgery, Birat Medical College Teaching Hospital, Tankisinuwari, Morang, Biratnagar 56613, Province 1, Nepal; Department of General Surgery, Birat Medical College Teaching Hospital, Tankisinuwari, Morang, Biratnagar 56613, Province 1, Nepal; Department of General Surgery, Birat Medical College Teaching Hospital, Tankisinuwari, Morang, Biratnagar 56613, Province 1, Nepal

**Keywords:** appendiceal neoplasms, appendix, case reports, cystic echinococcosis, Echinococcus granulosus, hydatid cyst, peritoneal diseases

## Abstract

Primary appendiceal hydatid disease is an exceptionally rare manifestation of cystic echinococcosis and may mimic appendiceal or gynecological malignancy, making preoperative diagnosis difficult. A 55-year-old postmenopausal woman presented with right iliac fossa pain and a palpable abdominal mass. Computed tomography demonstrated a calcified appendiceal mass with multiple peritoneal deposits and bilateral ovarian enlargement, suggestive of an appendiceal mucinous neoplasm with peritoneal dissemination. Diagnostic laparoscopy followed by laparotomy revealed lesions involving the appendix, peritoneum, omentum, right ovary, and uterine serosa. Complete excision of visible disease was performed using meticulous no-spillage techniques. Histopathological examination confirmed disseminated hydatid disease without evidence of neoplasia. Following postoperative management of complications, albendazole therapy was administered and the patient was discharged in stable condition. In endemic regions, hydatid disease should be considered in atypical appendiceal masses, even without hepatic or pulmonary involvement. Complete excision, histopathological confirmation, and postoperative antiparasitic therapy are important components of management.

## Introduction

Hydatid disease (cystic echinococcosis) is a zoonotic infection caused predominantly by the larval stage of Echinococcus granulosus. The liver and lungs are the most commonly affected organs, while primary extrahepatic and extrapulmonary disease is uncommon and may mimic inflammatory or neoplastic conditions [1–4]. Peritoneal and pelvic involvement are rare, with ovarian and uterine involvement being particularly uncommon [2, 5–10]. Appendiceal involvement is exceptionally rare and may mimic appendicitis, appendiceal abscess, mucocele, or neoplasm, with diagnosis often established only after histopathological examination [11–16].

We report an unusual case of primary appendiceal hydatid disease with disseminated involvement of the peritoneum, omentum, right ovary, and uterine serosa, presenting radiologically as an appendiceal neoplasm.

## Case presentation

A 55-year-old postmenopausal woman presented on 21 June 2026 with intermittent right lower abdominal pain for one month, associated with low-grade fever, intermittent non-bilious vomiting, and loss of appetite. She reported intermittent mild-to-moderate abdominal pain for ~4 years. Her surgical history included open myomectomy 25 years earlier, with no other significant medical history.

Repeated ultrasonography at other centers demonstrated bilateral bulky ovaries and a bulky fibroid uterus. On examination, she was hemodynamically stable with an Eastern Cooperative Oncology Group (ECOG) performance status of 1 and body mass index of 21 kg/m^2^. A firm-to-hard, non-tender ~6 × 6 cm mass was palpable in the right iliac fossa. Laboratory investigations showed normocytic anemia (hemoglobin 9.2 g/dL), while leukocyte count, eosinophil count, renal function tests, carcinoembryonic antigen (CEA), and cancer antigen 125 (CA-125) were largely within normal limits. Hepatic transaminases were mildly elevated (aspartate aminotransferase 48 U/L, alanine aminotransferase 72 U/L) (Table 1). Because hydatid disease was not clinically or radiologically suspected preoperatively, Echinococcus serology immunoglobulin G ELISA was not performed prior to surgery. Postoperative serological testing was also withheld given that complete surgical excision was achieved and histological confirmation had already established the diagnosis.

**Table 1:** Preoperative laboratory investigations demonstrating mild anemia and mildly elevated hepatic transaminases. Tumor markers (CEA and CA-125), leukocyte count, eosinophil count, renal function, bilirubin, alkaline phosphatase, and serum albumin were within normal limits

Parameter	Result	Reference range
Hemoglobin (g/dL)	9.2	12.0–16.0
Red blood cell count (×10^12^/L)	3.17	4.20–6.50
White blood cell count (×10^9^/L)	5.46	4.00–11.00
Neutrophils (%)	55	40–70
Eosinophils (%)	2	1–6
Platelet count (×10^9^/L)	336	140–440
Carcinoembryonic antigen (CEA) (ng/mL)	2.38	<5.0
Cancer antigen 125 (CA-125) (U/mL)	22.1	<35
Total bilirubin (mg/dL)	0.80	0.1–1.2
Direct bilirubin (mg/dL)	0.30	0.0–0.3
Aspartate aminotransferase (AST) (U/L)	48	5–45
Alanine aminotransferase (ALT) (U/L)	72	5–40
Alkaline phosphatase (ALP) (U/L)	92	66–240
Serum albumin (g/dL)	3.6	3.5–5.2
Blood urea (mg/dL)	12	10–40
Serum creatinine (mg/dL)	0.48	0.50–1.00

Preoperative laboratory investigations demonstrating mild anemia and mildly elevated hepatic transaminases. Tumor markers (CEA and CA-125), leukocyte count, eosinophil count, renal function, bilirubin, alkaline phosphatase, and serum albumin were within normal limits.

Abbreviations: ALP, alkaline phosphatase; ALT, alanine aminotransferase; AST, aspartate aminotransferase; CA-125, cancer antigen 125; CEA, carcinoembryonic antigen.

Contrast-enhanced computed tomography (CT) demonstrated an ill-defined 68.7 × 50.5 mm mildly enhancing lesion posterior to the cecum and ileocecal junction, containing coarse calcification with surrounding fat stranding and adjacent omental and mesenteric thickening (Fig. 1). Multiple calcified mesenteric and retroperitoneal nodules were also identified. Both ovaries were enlarged without solid components or mural nodules. No hepatic or pulmonary hydatid cysts were identified. The radiological impression favored a low-grade appendiceal mucinous neoplasm with peritoneal dissemination, with chronic appendiceal inflammatory pathology as an alternative diagnosis.

**Figure 1:**
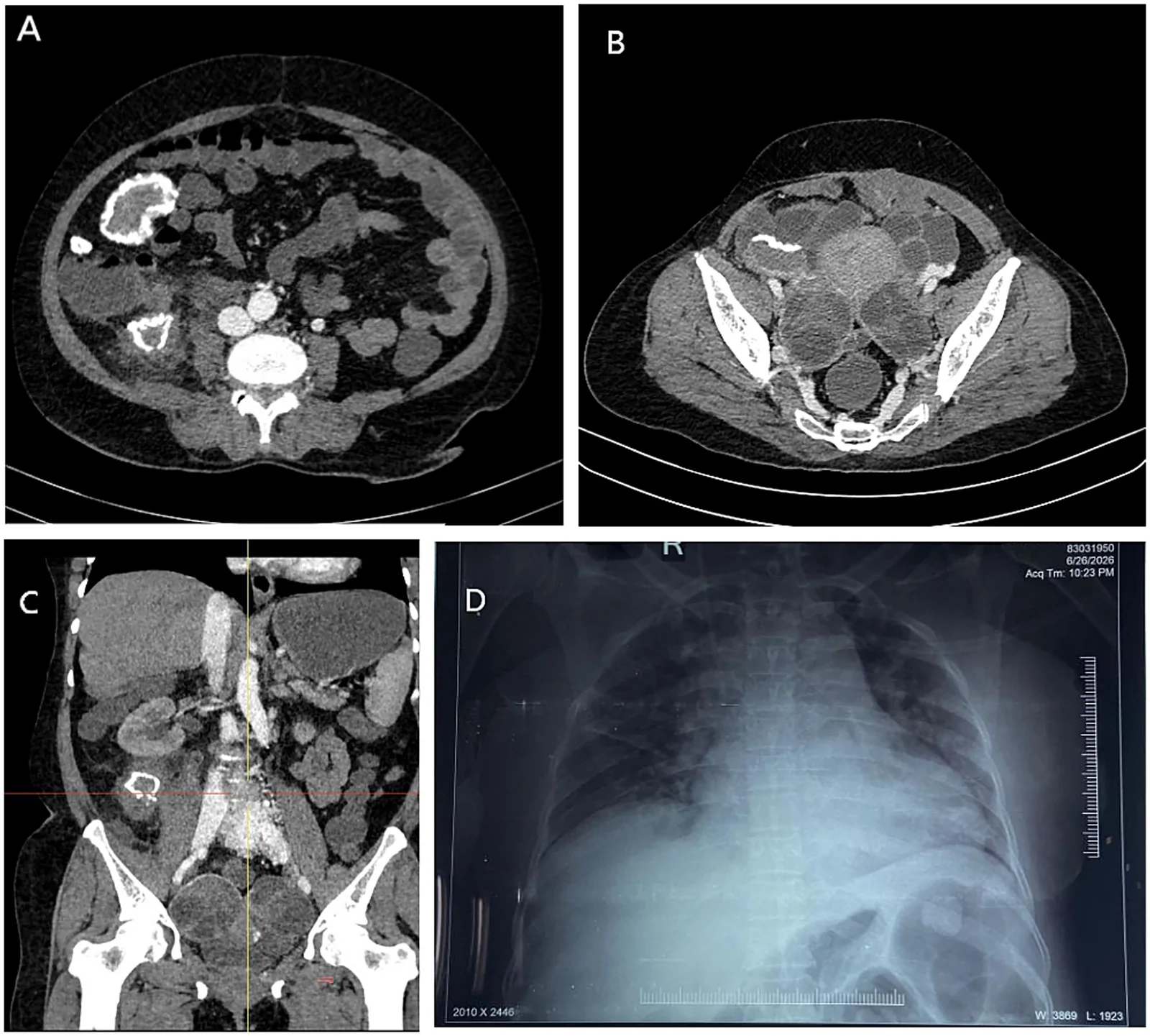
Contrast-enhanced CT images of the abdomen and pelvis and chest X-ray. (A) Axial section demonstrating a well-defined cystic lesion with peripheral calcification in the right lower quadrant, closely related to the ileocecal/appendiceal region. (B) Axial pelvic section showing multiple complex cystic lesions involving the bilateral adnexal regions. (C) Coronal reconstruction demonstrating the right lower abdominal calcified cystic lesion and associated complex pelvic lesions. (D) Chest radiograph demonstrating no pulmonary hydatid disease.

Diagnostic laparoscopy revealed multiple calcified and cystic lesions involving the appendix, right tubo-ovarian complex, parietal peritoneum, and greater omentum. Because of the extensive disease and the need to prevent cyst rupture and spillage, conversion to laparotomy was performed (Fig. 2). Following multidisciplinary consultation, total abdominal hysterectomy with bilateral salpingo-oophorectomy, appendectomy, and excision of all visible peritoneal and omental lesions were completed using meticulous no-spillage techniques. Because hydatid cyst dissemination was not suspected preoperatively, intraoperative scolicidal irrigation or hypertonic saline packing was not routinely applied prior to excision; however, strict surgical isolate protocols and gentle non-traumatic handling were maintained throughout the procedure. No intraoperative features of acute anaphylactoid reaction occurred.

**Figure 2:**
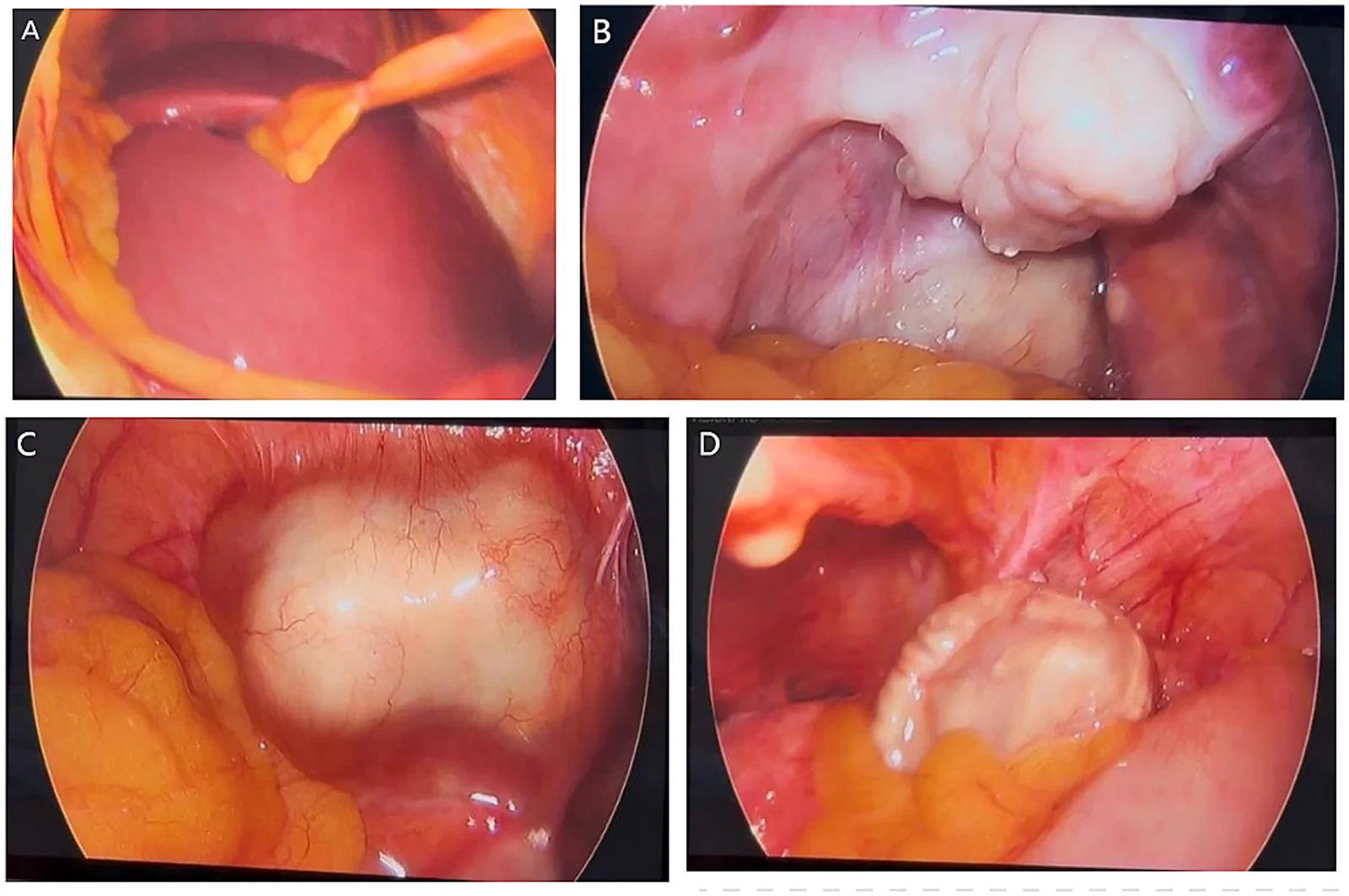
Intraoperative findings during diagnostic laparoscopy. (A) Smooth hepatic surface without visible hepatic cystic lesions suggestive of hydatid disease. (B) Lobulated whitish cystic/calcified lesion involving the right adnexal (tubo-ovarian) region. (C) Large, smooth-walled cystic lesion corresponding to the enlarged right ovary/adnexal mass. (D) Whitish nodular lesion involving the appendiceal/cecal region, corresponding to the appendiceal mass.

Gross examination showed an appendiceal specimen measuring 9 × 3.2 × 2.2 cm with an area of focal wall disruption containing thick gelatinous material, representing a pre-existing chronic micro-rupture or leak rather than intraoperative spillage. Similar calcified and gelatinous deposits were present in the omentum and peritoneum. One ovary contained a cystic lesion with thick gelatinous contents.

Histopathology demonstrated abundant acellular eosinophilic laminated ectocyst membranes with characteristic concentric, undulating, and folded lamellations, surrounded by fibrocollagenous tissue with mild chronic inflammation (Fig. 3). No epithelial lining, dysplasia, mucin-producing epithelium, or evidence of appendiceal mucinous neoplasia was identified. Similar laminated hydatid membranes were present in the peritoneal and omental deposits and on the ovarian and uterine serosal surfaces, confirming primary appendiceal hydatid disease with disseminated peritoneal, ovarian serosal, and uterine serosal involvement.

**Figure 3:**
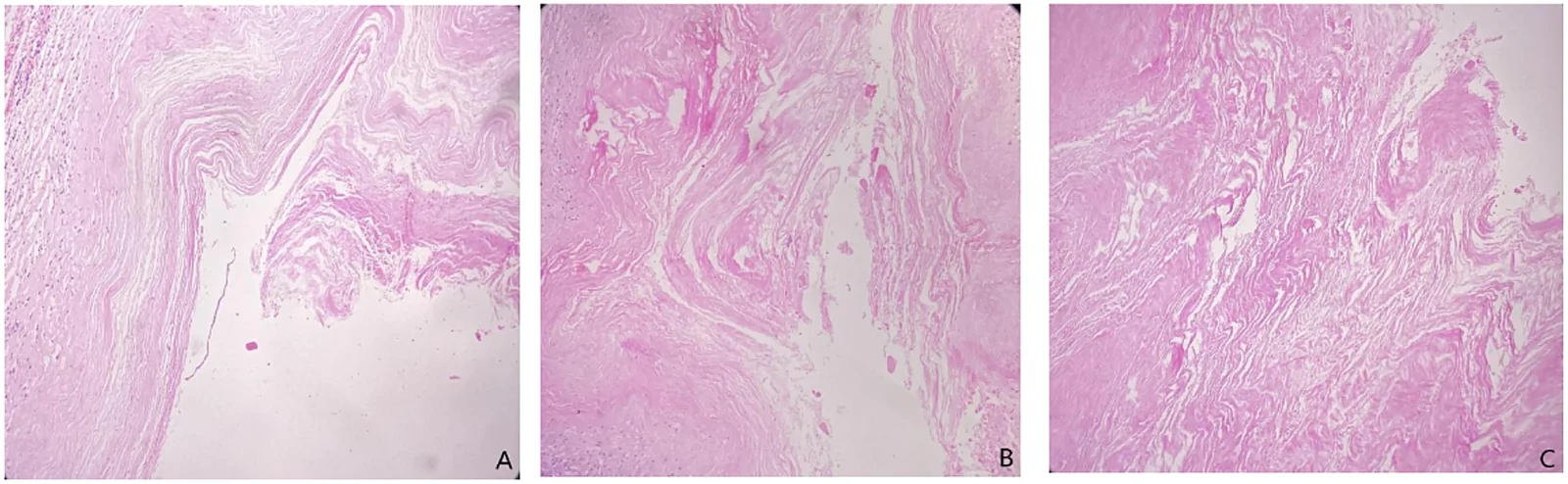
Histopathological findings of the resected lesions. (A) Low-power view demonstrating abundant acellular, eosinophilic, laminated ectocyst surrounded by fibrocollagenous host tissue with a mild chronic inflammatory infiltrate. (B) Folded and detached laminated membranes projecting into the cystic cavity, characteristic of cystic echinococcosis. (C) High-power view showing a thick, undulating, concentrically laminated ectocyst membrane, confirming hydatid disease. No evidence of malignancy is identified in the examined sections.

Following histopathological confirmation, epidemiological history was revisited. Although she denied direct dog contact, she reported daily exposure to cattle, buffaloes, and goats in a rural household environment.

The postoperative course was complicated by ileus, which resolved conservatively by postoperative day 3. A superficial surgical-site infection and subsequent bacteremia were successfully treated with culture-directed antibiotics. Albendazole 400 mg twice daily was initiated on postoperative day 10 and continued for four weeks (a standard 28-day cycle for localized/completely resected disease without ongoing cyst spillage). The patient was discharged in stable condition on postoperative day 14. At the 3-month follow-up evaluation, the patient remained asymptomatic, and follow-up abdominal ultrasonography demonstrated no evidence of disease recurrence or residual pelvic/peritoneal lesions.

## Discussion

Primary appendiceal hydatid disease is exceptionally uncommon and may mimic appendiceal malignancy, particularly when associated with calcified peritoneal deposits and pelvic organ involvement [3, 4, 11–16]. In the present case, the absence of hepatic or pulmonary disease supports a primary appendiceal origin. The extensive peritoneal, omental, ovarian, and uterine serosal involvement may reflect chronic leakage or rupture of the appendiceal cyst with subsequent intraperitoneal dissemination.

Preoperative diagnosis was diagnostic and challenging. From a radiological perspective, CT features such as an appendiceal mass with central/peritoneal calcifications, surrounding mucinous/gelatinous deposits, mesenteric stranding, and bilateral adnexal enlargement classic for Krukenberg-like spread heavily biased the differential toward a low-grade appendiceal mucinous neoplasm (LAMN) with pseudomyxoma peritonei or metastatic ovarian carcinoma. Furthermore, classic imaging hallmarks of hydatid cysts—such as multiloculated 'daughter cysts,' collapsed endocyst membranes ('water-lily sign'), or localized hepatic/pulmonary parenchymal cysts—were notably absent. Coupled with normal eosinophil counts and tumor markers (CEA, CA-125), these factors led the multidisciplinary team to favor a neoplastic etiology. Consequently, hydatid disease was not initially considered, and the true diagnosis was established only after surgical resection and histopathological examination.

Laparoscopy facilitated assessment of disease extent, while conversion to open surgery allowed complete excision of visible disease with meticulous avoidance of cyst spillage. Histopathology provided definitive confirmation through identification of the characteristic laminated ectocyst and exclusion of appendiceal mucinous neoplasia. In unusual anatomical locations, histopathological examination remains crucial when radiological findings are nonspecific.

Following confirmation, albendazole 400 mg twice daily was administered for four weeks. While prolonged therapy is often warranted for incomplete resection or intraoperative spillage, a single 4-week cycle was considered appropriate following complete en bloc macroscopic resection. Given the disseminated nature of her initial presentation, long-term surveillance with clinical evaluations and interval abdominal imaging at 6 and 12 months is planned to ensure early detection of any late recurrence.

## Conclusion

Primary appendiceal hydatid disease with disseminated peritoneal, ovarian, and uterine serosal involvement is an exceptionally rare presentation of cystic echinococcosis. In endemic regions, hydatid disease should be considered in the differential diagnosis of calcified appendiceal masses, even without hepatic or pulmonary involvement. Careful surgical handling to prevent spillage, complete excision of visible disease, histopathological confirmation, and postoperative antiparasitic therapy are important for optimal management.

## Reporting statement

This manuscript was prepared and structured following the CARE guidelines and case reporting standards.
